# How Can Non-Hospital Surgical Centres Improve Their Environmental Footprint (and Reduce Costs)?

**DOI:** 10.1177/22925503241305635

**Published:** 2025-01-03

**Authors:** Barinder Bajwa, Zach Zhang, Young Ji Tuen, Rebecca Courtemanche, Jugpal S. Arneja

**Affiliations:** 1 Division of Plastic Surgery, Department of Surgery, 8166 University of British Columbia, Vancouver, BC, Canada; 2 Sauder School of Business, 12358 University of British Columbia, Vancouver, BC, Canada

**Keywords:** non-hospital surgical centre, carbon footprint, operating room, anesthetic gases, disposable equipment, cost benefit, centre chirurgical non hospitalier, empreinte carbone, salle d’opération, gaz anesthésiques, matériel jetable, coût-avantages

## Abstract

**Introduction:**

Every industry has greenhouse gas emissions, with healthcare a significant contributor. In Canada, the healthcare sector is directly and indirectly responsible for 4.6% of the country's greenhouse gas emissions. Operating rooms (ORs) are major contributors to hospital waste, making the OR low hanging fruit for analyzing environmental practices. The OR can adopt a green mindset to reduce its carbon footprint, yet barriers to going green exist. Herein we study non-hospital surgical centres in British Columbia to assess current green practices, attitudes towards environmental sustainability, and barriers to implementation.

**Methods:**

All accredited non-hospital surgical centres in BC were invited to complete a survey on current practices and plans to reduce their environmental impact.

**Results:**

Of 56 non-hospital surgical centres contacted, 18 responded, with 89% willing to adapt their practice to promote environmental sustainability, yet lacked current knowledge (56%) and formal plans (0%). The wide use of anesthetic gases with high global warming potential (64%) and disposable drapes/ gowns (78%/ 67%) were noted. Barriers to adopting green practices included: cost (44%), infrastructure (44%), regulatory guidelines (39%), knowledge (39%), and safety (28%).

**Conclusions:**

Transitioning to more environmentally sustainable practices in ORs can enhance healthcare value by reducing both costs and greenhouse gas emissions. The greatest effect can be achieved through prudent choice of anesthetic gas agent, followed by reusable linens and drapes. Education and regulatory leadership were identified as crucial for overcoming these barriers. This study underscores the need for education, guidelines, and economically viable options to transition from awareness to action.

## Résumé

**Introduction:**

Ttoutes les industries émettent des gaz à effet de serre, et le milieu de la santé y contribue énormément. Au Canada, le secteur de la santé est directement et indirectement responsable de 4,6% des émissions de gaz à effet de serre du pays. Les salles d’opération (SO) contribuent largement aux déchets hospitaliers, ce qui en fait le milieu idéal pour analyser les pratiques environnementales. Les SO peuvent adopter une optique écologique pour réduire leur empreinte carbone, mais il y a des obstacles à devenir vert. Dans le présent article, les auteurs étudient les centres chirurgicaux non hospitaliers de la Colombie-Britannique pour évaluer les pratiques écologiques en place, les attitudes envers la durabilité environnementale et les obstacles à leur mise en œuvre.

**Méthodologie:**

Tous les centres chirurgicaux non hospitaliers agréés de la Colombie-Britannique ont été invités à remplir un sondage sur les pratiques et plans en place pour réduire leur impact environnemental.

**Résultats:**

Des 56 centres chirurgicaux non hospitaliers contactés, 18 ont répondu, et 89 % étaient prêts à adapter leur pratique afin de promouvoir la durabilité environnementale, mais n’avaient pas de connaissances à jour (56 %) ou de plans officiels (0 %). La vaste utilisation des gaz anesthésiques au fort potentiel de réchauffement mondial (64 %) ainsi que des champs opératoires et des blouses jetables (78 % et 67 %) a été soulignée. Les obstacles à l’adoption de pratiques vertes incluaient les coûts (44 %), infrastructure (44 %), les directives en matière de réglementation (39 %), les connaissances (39 %) et la sécurité (28 %).

**Conclusions:**

La transition vers des pratiques environnementales plus durables en SO pourrait améliorer la valeur des soins grâce à la diminution des coûts et des émissions de gaz à effet de serre. Pour obtenir le maximum d’effet, il faut faire un choix prudent d’agent anesthésique et adopter de la literie et des champs stériles réutilisables. La formation et le leadership en matière de règlementation ont été déterminés comme essentiels pour vaincre ces obstacles. La présente étude fait ressortir la nécessité de prévoir des formations, des lignes directrices et des options viables sur le plan économique pour passer de la sensibilisation à l’action.

## Introduction

A warming planet is a worldwide concern and all industries are greenhouse gas emitters, which contribute to the changing climate, including healthcare.^1^ Canada's healthcare sector contributes 4.6% of the country's overall greenhouse gas (GHG) emissions.^2^ The healthcare industry often disposes of commonly recyclable materials such as plastic, paper and metal as general waste, leading to environmental harm primarily through treatment processes like landfilling and incineration. In particular, operating rooms (ORs) can contribute up to 50% of hospital waste and certain procedures may accumulate up to 50 pounds of waste.^3,4^ With the OR being a crucial yet resource-intensive aspect of healthcare facilities, it is a practical place to analyze practices in order to influence the carbon footprint of healthcare.

Healthcare facilities can undertake several evidence-based green practices, such as choosing anesthetic gases based on their environmental footprint, reducing energy consumption by utilizing LED lights, minimizing unnecessary devices and packaging, and opting for reusable items.^5,6^ However, barriers to implementing green practices exist, particularly in institutions such as hospitals. These barriers include manufacturer restriction, lack of support from leadership, and inadequate education.^7,8^ Conversely, institutional barriers may be lower in non-hospital surgical centres, where medical directors have a greater capacity to enact measures. In British Columbia (BC), guidance and facility accreditation are administered by The College of Physicians and Surgeons of BC (CPSBC) Non-Hospital Medical and Surgical Facilities Accreditation Program (NHMSFAP).

Currently, we are not aware of green practice policies or barriers that exist within non-hospital surgical centres in BC. This study aims to determine current green practices, as well as attitudes towards and barriers to environmental sustainability initiatives, in non-hospital surgical centres across BC. We hope these findings can provide a province-wide snapshot of current practices and efforts toward environmental sustainability. These results may inform future environmental sustainability programs and educational resources for facilities and the NHMSFAP.

## Methods

This study was designed as an anonymous survey which was distributed to all non-hospital surgical centres in BC. Ethics approval was obtained from the University of British Columbia Children’s and Women’s Health Centre of British Columbia Research Ethics Board (H22-01257). Invitation letters and survey were mailed to facilities, and the survey remained open from July 2022 to February 2023.

### Study Design

The survey questions (Supplemental Information 1) were modified based on previous published studies.^7–9^ The survey also asked questions about the respondent's background, knowledge, current practices (energy and hydro, anesthetic, waste management), and current barriers to move towards environmentally-friendly practices. The survey questions were a combination of Likert scale, multiple choice, and open-ended questions. 56 surgical centres in BC were invited to participate in this study.

#### Data Analysis

Data were summarized and tabulated in Microsoft Excel and analyzed descriptively with counts and frequencies.

## Results

### Demographics of Respondents

The survey was distributed to 56 non-hospital surgical centres across BC, and we obtained 18 responses (32% response rate). Among the participants, 94% (17 individuals) were medical directors, while the remaining 6% (1 individual) was a facility manager. With regards to facility size, 9 centres (50%) had a single operating room, 5 centres (28%) had two, 1 centre (6%) had three, 1 centre (6%) had four, and 2 centres (11%) had more than five operating rooms in their surgical facility, Table 1. Concerning annual procedure volumes, 1 centre handled less than 250 procedures, 4 centres handled procedures ranging from 250 to 500, 1 centre handled procedures ranging from 501 to 750, 3 centres handled procedures ranging from 751 to 1000, and 9 centres were involved in more than 1000 procedures. Finally, the most common users of the centres were Plastic Surgery (33%, 6 centres), Ophthalmology (28%, 5 centres), Obstetrics and Gynecology (22%, 4 centres), Orthopedic Surgery (17%, 3 centres), Dermatology (6%, 1 centre), General Surgery (6%, 1 centre), and Vascular Surgery (6%, 1 centre).

**Table 1.** Demographics and Attitudes Regarding Environmental Sustainability.

PARAMETERS	ALL RESPONDENTS (N = 18)
**Demographics**	
**Respondent, n (%)**	
Medical director	17 (94)
Facility manager	1 (6)
**Facility size, n (%)**	
1 operating room	9 (50)
2 operating rooms	5 (28)
3 operating rooms	1 (6)
4 operating rooms	1 (6)
≥5 operating rooms	2 (11)
**Annual procedure volume, n (%)**	
<250	1 (6)
250–500	4 (22)
501–750	1 (6)
751–1000	3 (17)
>1000	9 (50)
**Most common specialty type user of facility, n (%)**	
Plastic surgery	6 (33)
Ophthalmology	5 (25)
Obstetrics and gynecology	4 (22)
Orthopedic surgery	3 (17)
Dermatology	1 (6)
General surgery	1 (6)
Vascular surgery	1 (6)
**Attitudes Regarding Environmental Sustainability**	
**Environmental impact of surgery is important, n (%)**	
Strongly agree/ agree	13 (72)
Uncertain	2 (11)
Disagree/ strongly disagree	3 (17)
**Willingness to adapt practice to promote environmental sustainability, n (%)**	
Strongly agree/ agree	16 (89)
Uncertain	2 (11)
Disagree/ strongly disagree	0
**Sufficient level of knowledge regarding environmental sustainability, n (%)**	
Strongly agree/ agree	8 (44)
Uncertain	5 (28)
Disagree/ strongly disagree	5 (28)
**Education on environmental sustainabilty**	3 (17)
**Written plan to reduce carbon footprint, n (%)**	0 (0)
**Net zero carbon neutral target, n (%)**	0 (0)
**Sustainability lead in place, n (%)**	1 (6)

### Attitudes Regarding Environmental Sustainability

Most respondents (13/18, 72%) indicated that they strongly agree/agree that the environmental impact of surgery is important, Table 1. Furthermore, the majority (16/18, 89%) indicated their willingness to adapt their practices to promote environmental sustainability. However, over half of the respondents (10/18, 56%) expressed a lack of confidence (uncertain or disagree/strongly disagree) that they had a sufficient level of knowledge regarding environmental sustainability.

Regarding education and training on environmental sustainability, a majority (15/18, 83%) reported never having received any form of instruction in this area, Table 1. Furthermore, none of the respondents (0/18, 0%) had a written plan to reduce their surgical centre's carbon footprint or a carbon neutral target. In addition, 94% (17/18) of the surgical centres surveyed lacked a designated sustainability lead.

### Green Energy and Hydro Practices

A majority of the respondents (14/18, 78%) utilize LED lighting within their non-hospital surgical centres, Table 2. Additionally, all respondents (18/18, 100%) reported a practice of turning off the lights in the operating room during nights and weekends. However, almost all respondents (17/18, 94%) do not have occupancy sensors installed in their surgical centre. Similarly, a majority (13/18, 72%) reported that their HVAC (heating, ventilation, and air conditioning) systems do not automatically turn off when not in use. Regarding hydro practices, 44% (8/18) of the respondents reported implementing waterless surgical scrub solutions. Among those who do use water, most respondents (15/18, 83%) have automatic sensors to turn off the tap between hand washing.

**Table 2.** Current Practices and Barriers to Environmental Sustainability.

PARAMETERS	ALL RESPONDENTS (N = 18)
**Green Energy and Hydro Practices**	
LED lighting used, n (%)	14 (78)
Turning off lights in OR during nights and weekends, n (%)	18 (100)
Occupancy sensors, n (%)	1 (6)
HVAC automatic shut-off, n (%)	5 (28)
Waterless surgical scrub solutions, n (%)	8 (44)
Automatic handwashing water tap shut-off, n (%)	15 (83)

**Anesthetic Gases**	
Anesthetic gases used, n (%) Sevoflurane Desflurane Nitrous oxide	5 (36) 5 (36) 4 (29)
Low-flow anesthetic policy, n (%)	2 (13)
Gas recapture system, n (%)	0 (0)

**Waste Management Practices & Linen Utilization**	
Waste separation program, n (%)	14 (78)
Most commonly recycled materials, n (%) Paper Cardboard Rigid plastic containers Soft plastic wrappers Glass Metal	17 16 13 10 9 4
No preference for disposable over sterilizable instruments, n (%)	15 (83)
Single-use products, n (%) Drapes Surgical gowns Anesthetic mask Monopolar cautery Padding Other^a^ Laryngoscope Blood pressure cuff	14 (78) 12 (67) 6 (33) 5 (28) 2 (11) 2 (11) 0 (0) 0 (0)
Waste management practices, n (%) Recycling non-contaminated waste Reducing single use instrument Reducing IV fluid use Reusing surgical gowns Redesigning trays Reusing surgical drapes Switching blue sterile tray wraps Reducing plastic syringe use Reducing disposable glove use Minimizing packaging for products	13 (72) 10 (55) 7 (39) 6 (33) 5 (28) 3 (17) 2 (11) 2 (11) 1 (6) 0 (0)

**Barriers**	
Barriers to improving environmental sustainability, n (%) Available infrastructure Cost Lack of knowledge Logistics Safety Staff attitudes Lack of support from colleagues/leadership *Other/Comments about barriers* CPSBC NHMSFAP Lack of alternatives for single use products Impractical infection control and reprocessing fears	8 (44) 8 (44) 7 (39) 7 (39) 5 (28) 3 (17) 2 (11) *9 (50%)* 7 (39%) 1 (6%) 1 (6%)

OR, operating room; ^a^other: suction tubing, gloves, electrodes; HVAC, heating, ventilation, and air conditioning; CPSBC NHMSFAP The College of Physicians and Surgeons of BC Non-Hospital Medical and Surgical Facilities Accreditation Program

### Anesthetic Gases

The types of gases used across centres in BC included sevoflurane, desflurane and nitrous oxide, Table 2. Most respondents (9/14, 64%) used desflurane or nitrous oxide (gases with high global warming potential). In our survey, 2 (13%) of respondents stated that their non-hospital centre implemented a low-flow anesthetic policy. Furthermore, 100% of the participants reported that their facilities did not have a gas recapture system.

### Waste Management Practices and Linen Utilization

A majority of respondents (14/18, 78%) reported having a waste separation program, Table 2. The most commonly recycled materials were paper, cardboard, and rigid plastic containers. In addition, most (15/18, 83%) reported having no preference for disposable instruments over sterilizable ones, yet single-use products such as drapes, and surgical gowns were the predominant choice in 78% and 67% of centres, respectively. Waste management practices are highlighted in Table 2.

### Barriers

Cost and safety were the greatest concerns against the adoption of reusable instruments and products, Table 2. Available infrastructure, cost, and logistics were highlighted as barriers to recycle. Furthermore, 39% of participants mentioned the CPSBC NHMSFAP guidelines as a barrier to implementing new practices.

## Discussion

The environmental impact of non-hospital surgical centres in BC remains largely unknown. The results of this study provide insight into existing practices, attitudes, and barriers pertaining to environmental sustainability efforts in non-hospital surgical centres across the region. Although these surgical centres are doing well in some domains, the results of our study reveal an intriguing pattern: while there is some awareness and clear motivation to embrace eco-friendly practices, there is an absence of existing initiatives, plans, or regulatory guidance. This transition to action is limited by formal education, guidelines, and knowledge around existing economically viable options.

### Anesthetic Gases

Commonly used anesthetics such as, desflurane, and nitrous oxide hold considerable implications for environmental pollution due to their greenhouse gas effects. Desflurane's capacity to capture thermal energy within the Earth's atmosphere is nine times greater than that of nitrous oxide and twenty times greater than that of sevoflurane.^10^ Furthermore, desflurane can linger in the atmosphere for 14 years, much longer than sevoflurane's lifetime of 1.1 years.^10,11^ Several studies have demonstrated that sevoflurane is equivocal to desflurane in terms of postoperative major morbidities or mortality, hemodynamic variables, postoperative pain scores, and emergence agitation.^12,13^ Considering its comparable efficacy and smaller environmental footprint, sevoflurane is the clear green winner compared to desflurane. Moreover, research has shown significant environmental and economic benefits of reducing the use of harmful anesthesic gases in healthcare. Hensher et al, discuss the feasibility and importance of incorporating environmental costs, such as the social cost of carbon, into health economic evaluations, reinforcing the need for sustainable anesthetic practices.^14^

In comparison to desflurane and sevoflurane, nitrous oxide has an atmospheric lifetime of approximately 114 years.^10,11^ Notably, nitrous oxide, while being one of the least potent inhaled anesthetics, lacks the ability to reliably induce general anesthesia when used in isolation, so it is often combined with sevoflurane or other more potent anesthetics. Yet, concerns have been raised about the use of nitrous oxide, as studies have demonstrated combining nitrous oxide with sevoflurane does not provide a significant anesthetic advantage in terms of efficacy, safety or onset, yet nitrous oxide increases greenhouse gas emissions when combined with other agents.^15–17^

From an economic perspective, desflurane has higher cost profile as compared to that of the cheaper sevoflurane.^18^ In a study conducted by Tabing et al, desflurane was calculated to incur a cost of $13.20 per case, while sevoflurane amounted to $0.63 per case.^19^ Extrapolating from our survey finding of 250 to 1000 annual cases, transitioning from desflurane to sevoflurane could potentially save $3200 to $13,000 annually per centre. Given the above evidence, in 2022, the American Society of Anesthesiologists recommended the removal of desflurane from drug formularies. Furthermore, they recommend decommissioning central nitrous oxide piping and avoidance of nitrous oxide use.^20^ As of March of 2023, Scotland has become the first country to ban desflurane and England will soon introduce a similar ban in 2024.^21^ Overall, to address the financial burden associated with costly drugs such as desflurane, interventions aimed at reducing their accessibility have demonstrated the potential for substantial cost reductions without compromising patient safety or recovery.^19^ Taken together, the environmental, safety, and economic benefits strongly advocate for the transition from desflurane and nitrous oxide to more sustainable anesthetic practices. By reducing the use of these environmentally harmful and expensive agents, healthcare facilities can reduce their economic burden while preserving patient safety and improving their carbon footprint.

### Linens and Textiles

Despite the majority (83%) of respondents in our survey expressing no preference for disposable over sterilizable instruments, 78% of centres still utilize disposable drapes and 67% use disposable gowns.

Existing literature widely supports the environmental advantages of reusable textiles. For instance, switching to reusable gowns can lead to a significant reduction in natural resource and energy consumption (by 64%), greenhouse gas emissions (by 66%), blue water consumption (by 83%), and solid waste generation (by 84%).^5^ Unger et al also found that reusable medical products generally result in lower environmental and economic costs compared to disposable alternatives.^22^ Our study identified safety and cost as the primary concern against the adoption of greener reusable products. A study by McQuerry et al found superior protection and consistent fabric and seam strength in reusable gowns throughout their expected lifespan, irrespective of brand or protection level.^23^ The mixed-methods study by Yap et al highlighted that while most perioperative staff recognized the environmental benefits of reusable gowns, concerns about their functionality, comfort, and cost remained significant barriers to adoption.^24^

Additionally, the life cycle assessment conducted by Campion et al on disposable custom packs revealed that disposable materials contribute significantly to healthcare waste, accounting for approximately 33 pounds of waste per patient bed per day.^25^ The study highlighted that polypropylene and cotton were the most prominent materials, with cotton having the largest environmental impact across all environmental impact categories.

The cost profile of sterile versus reusable OR gowns depends on multiple factors, such as the healthcare facility, the frequency of surgical procedures, and the specific reusable gown program in place. Several studies have demonstrated substantial savings through the transition to reusable gowns. A study conducted in 2024 demonstrated that replacing disposable gowns with reusable alternatives led to a 66% decrease in carbon footprint, alongside annual cost savings of £13 483.89.^26^ Another study showed savings of nearly 50% per gown use after switching from disposable ($0.79 per use) to reusable gowns ($0.39 per use).^27^ Another study in Turkey revealed reusable drapes and gowns to be more cost-effective, reporting a cost weight of 0.094 for reusable sets versus 0.906 for single-use sets.^28^ Variations in cost outcomes can be influenced by factors like local contract negotiations with suppliers, manufacturers, or distributors. Through adoption of reusable textiles and linens, facilities can expect a significant decrease in their carbon footprint and a potential reduction of costs, while maintaining patient safety.

### Education and Leadership

Lack of knowledge is widely recognized as a major barrier to adopting environmentally friendly practices. Research by Azouz et al demonstrated a 10.3% monthly cost savings after implementing an education program in which an education module and training regarding OR waste disposal was provided to staff.^9^ Another study by Wormer et al highlighted the benefits of having a dedicated Green OR committee and their successful efforts in diverting medical waste (6.5 tons) and saving over $50 000 annually through the adoption of reusable gel pads.^4^

Higher education institutions also play a pivotal role in promoting sustainability and climate action. They provide education, conduct relevant research, and implement sustainable campus operations. According to Filho et al, these efforts are crucial in developing the next generation of environmentally conscious healthcare professionals.^29^

Our survey results indicate the main barriers to improving environmental sustainability are available infrastructure, cost, lack of knowledge, and logistics. Overcoming these barriers and implementing evidence-based green initiatives can be achieved by educating relevant stakeholders such as surgeons and medical directors about the carbon footprint of operating rooms and creating awareness that alternative solutions exist. The CPSBC NHMSFAP can be an educational partner and provide policy guidance on best practices.

In addition to education on environmental sustainability, there are several actionable steps that medical directors of non-hospital surgical centres can implement. For instance, pre-existing initiatives like Practice Greenhealth's “Greening the OR” program provide a wealth of resources and tools for sustainable practices such as the Greenhealth reusable gowns initiative.^30^ Collaborating with governmental bodies and professional organizations such as the College of Physicians and Surgeons of British Columbia (CPSBC) can offer additional support and guidance. Useful resources include the BC Green Care initiative, the BC Climate Action Toolkit, and the nitrous oxide project, all of which provide a framework for reducing emissions associated with medical gas management.^31–33^ Additionally, the Canadian Medical Association (CMA) offers insights into achieving a net-zero health system, which can be instrumental in developing a ‘green toolkit’ for surgical centres.^34^ By leveraging these strategies and resources, medical directors can make substantial progress in reducing the environmental impact of their facilities.

### Limitations

This is the first study on self-reported environmental practices of non-hospital surgical centres in BC. This study was limited by self-respondent bias, recall bias, and response rate, as individuals possessing greater knowledge on the subject might display a higher inclination to participate. Conducting economic comparisons for anesthetics and linen presented a distinct challenge due to the involvement of various specific factors such as geographical differences, brand, type, quality, and supplier which makes quantitative comparisons challenging. For example, the cost of anesthetics can vary across regions, as well as based on the utilization and duration of surgery. Although we have used the terms carbon footprint, environmental footprint, environmental sustainability, environmental impact, and greenhouse gas emissions interchangeably, we recognize these terms may be distinctive within the field of sustainability science and encompass different environmental impact categories.^35^

## Conclusion

Michael Porter has defined healthcare value as outcome divided by cost.^36^ Thus, value can be improved by either improving outcome or reducing cost, or through a combination of both. Our study found most non-hospital surgical centres were open to improving their environmental impact. This may be achieved most easily by minimizing the use of desflurane and nitrous oxide and promoting the use of reusable linens in outpatient settings, thus reducing cost and thereby improving healthcare value. Collaborating with other healthcare professionals, such as anesthesiologists, as well as looking to other regions that have already implemented such changes can help us determine the feasibility of our suggestions in our local region. Future work should focus on identifying and overcoming other obstacles to implementing our recommendations such as: encouraging and working with the CPSBC to consider NHMSFAP guidelines from an environmental perspective and providing education to medical directors around environmentally friendly practices. Additionally, studying the environmental impact in other provinces, and comparing attitudes and practices, can provide valuable insights and foster mutual learning.

## Study-Related Presentations

This work was presented at the Western Medical Research Conference, Carmel, CA, January 2023.

## Supplemental Material

sj-docx-1-psg-10.1177_22925503241305635 - Supplemental material for How Can Non-Hospital Surgical Centres Improve Their Environmental Footprint (and Reduce Costs)? Supplemental material, sj-docx-1-psg-10.1177_22925503241305635 for How Can Non-Hospital Surgical Centres Improve Their Environmental Footprint (and Reduce Costs)? by Barinder Bajwa, Zach Zhang, Young Ji Tuen, Rebecca Courtemanche and Jugpal S. Arneja in Plastic Surgery
