# Supplementary material for: How Can Non-Hospital Surgical Centres Improve Their Environmental Footprint (and Reduce Costs)?
Source: Plast Surg (Oakv). 2025 Jan 3;33(3):419–26. doi: 10.1177/22925503241305635 (PMC11696936; doi:10.1177/22925503241305635)
Supplement: sj-docx-1-psg-10.1177_22925503241305635 - Supplemental material for How Can Non-Hospital Surgical Centres Improve Their Environmental Footprint (and Reduce Costs)? [file sj-docx-1-psg-10.1177_22925503241305635.docx]

**SUPPLEMENTAL INFORMATION 1: SURVEY QUESTIONS**

**Background**

1. What is your role in the surgical center?
   - Medical Director
   - Facility Nursing Director
   - Director of perioperative services
   - Medical Office Assistant
   - Resident
   - Surgeon
   - Other
2. How many functional operating rooms does the center have?
   - 1
   - 2
   - 3
   - 4
   - ≥5
3. How many procedures occur at the center annually?
   - <250
   - 250-500
   - 501-750
   - 751-1000
   - >1000
4. What are the top 3 surgical specialties with highest surgical volume at the surgical center?
   - Plastic surgery
   - Ophthalmology
   - Dermatology
   - Orthopedic surgery
   - General surgery
   - Urology
   - Vascular surgery
   - Gynecology/ obstetric

**Knowledge**

1. To what extent do you agree with the following statement: The environmental impact of surgical procedures is an important factor that should be considered when conducting surgeries
   - Strongly disagree
   - Disagree
   - Uncertain
   - Agree
   - Strongly Agree
2. To what extent do you agree with the following statement: My level of knowledge on the environmental impact of surgical related agents, products and procedures is sufficient to guide the surgical facility to be more environmentally sustainable
   - Strongly disagree
   - Disagree
   - Uncertain
   - Agree
   - Strongly Agree
3. To what extent do you agree with the following statement: I am willing to change the practice at my workplace to reduce our environmental carbon footprint.
   - Strongly disagree
   - Disagree
   - Uncertain
   - Agree
   - Strongly Agree
4. Does your facility have a written plan to reduce the carbon footprint of your facility?
   - Yes
   - No
   - I don’t know
   - I prefer not to answer
5. Does your facility have a sustainability lead in place?
   - Yes
   - No
   - I don’t know
6. Have you received any training/education on environmental sustainability?
   - Yes
   - No
7. *If answer to 10 is yes –* What is the format of environmental education?
   - Workshop
   - Conference
   - Online module
   - Independent reading
   - Other: Comment box

________________________________________________________________________

1. Does your facility have a target to become ‘net zero’ carbon neutral?
   - Yes
   - No
   - Other: comment

________________________________________________________________________

**Current Practice**

**Energy & Hydro**

1. Does your workplace have an active plan to reduce the environmental impact of operating theatres and surgeries?
   - Yes
   - No
   - I don’t know
2. Does your facility use LED (light – emitting diode) for lighting?
   - Yes
   - No
   - I don’t know
3. Does your facility use occupancy sensors?
   - Yes
   - No
   - I don’t know
4. Does your facility turn off the lights in the operating theatre at nights and weekends?
   - Yes
   - No
   - I don’t know
5. Does your facility turn off the HVAC when not in use?
   - Yes
   - No
   - I don’t know
6. Does your facility use waterless surgical scrub solution?
   - Yes
   - No
   - I don’t know
7. Does your facility switch off taps between hand washing?
   - Yes
   - No
   - I don’t know

**Anesthetic**

1. What percentage of procedures at your facility use inhaled anesthetic (i.e. desflurane, isoflurane, sevoflurane, halothane)?
   - <20%
   - 21-40%
   - 41-60%
   - 61-80%
   - 81-100%
2. Please check all the inhaled anesthetic agents used at your surgical facility
   - Desflurane
   - Isoflurane
   - Sevoflurane
   - Halothane
   - Nitrous oxide
   - Xenon
   - Other: *Comment*

________________________________________________________________________

1. What is the most common inhaled anesthetic agent at your surgical facility?
   - Desflurane
   - Isoflurane
   - Sevoflurane
   - Halothane
   - Nitrous oxide
   - Xenon
   - Other: *Comment*

________________________________________________________________________

1. For the following questions: please check all the options that apply to your facility.

- The facility is reducing desflurane use
- The facility is eliminating desflurane from the operating theatre
- The facility is reducing nitrous oxide use
- The facility is eliminating nitrous oxide use
- Instead of inhaled anesthetic gases, the facility mandating the use of total intravenous anesthesia

1. Does your surgical facility have a low-flow anesthetic policy?
   - Yes
   - No
   - I don’t know
2. Do your operating room(s) use gas recapture system?
   - Yes
   - No
   - I don’t know

**Waste Management**

1. Does your facility have a waste separation program?
   - Yes
   - No
   - I don’t know
2. Does your facility prefer disposable instruments over re-sterilizable instruments?
   - Yes
   - No
   - I don’t know
3. What products in your facility is single use? Please check all that apply:
   - Surgical Gowns
   - Drapes
   - Anesthetic mask
   - Laryngoscope
   - Blood pressure cuff
   - Monopolar cautery
   - Padding
   - Other: *Comment*

________________________________________________________________________

1. What wastes are recycled in the OR? Please check all that apply:
   - Soft plastic wrappers
   - Rigid plastic containers
   - Glass
   - Paper
   - Cardboard
   - Metal
   - Other: *Comment*

________________________________________________________________________

1. Please choose the waste management practices that are being implemented at your facility. Please check all that apply
   - Recycling non-contaminated waste
   - Reducing plastic syringe use
   - Reducing unnecessary IV fluid/drug use
   - Reducing single use instrument
   - Reducing disposable glove use
   - Reusing surgical gowns
   - Reusing surgical drapes
   - Minimizing packaging for products
   - Redesigning trays for organization (ex. smaller sets)
   - Switching blue sterile tray wraps to meal canister trays

**Barriers**

1. What is the greatest barrier to using reusable products at your facility?
   - Convenience
   - Cost
   - Available infrastructure
   - Safety
   - Other: *Comment*

________________________________________________________________________

1. What is the greatest barrier to recycling at your facility?
   - Convenience
   - Cost
   - Available infrastructure
   - Safety
   - Other: *Comment*

________________________________________________________________________

1. Which of the following are barriers to improving environmental sustainability at your facility? Please check all that apply:
   - Lack of knowledge
   - Staff attitudes
   - Lack of support from colleagues and/or leadership
   - Safety concerns
   - Available infrastructure
   - Cost
   - Logistics
   - Other: *Comment*

___________________________________________________________________

1. Please use the following space to leave any comments or questions that were not addressed within this survey questionnaire.

____________________________________________________________________________

____________________________________________________________________________

____________________________________________________________________________

____________________________________________________________________________

____________________________________________________________________________

____________________________________________________________________________
